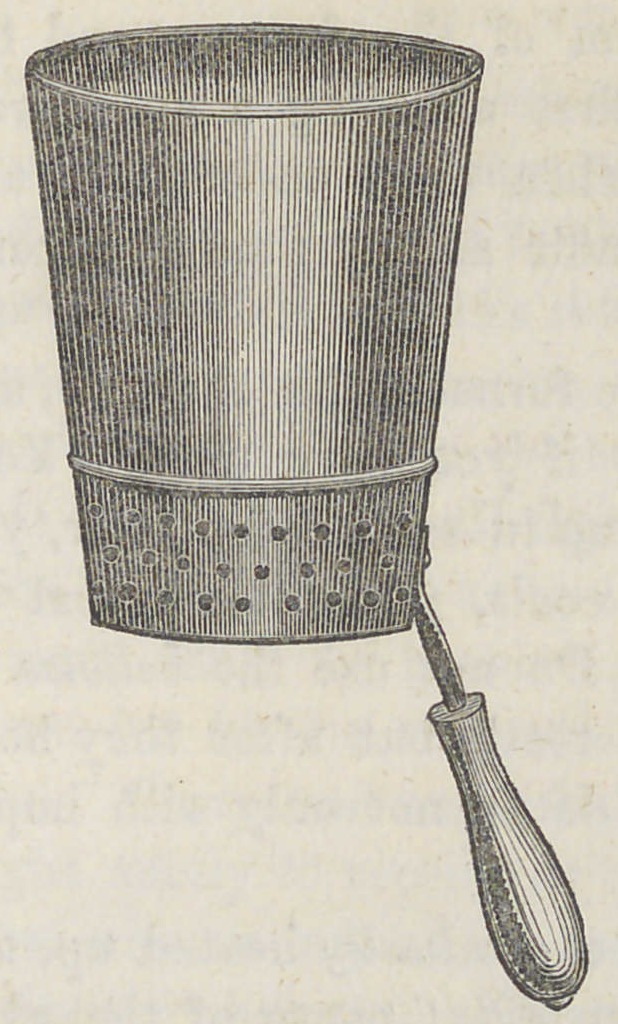# Soldering Made Easy

**Published:** 1859-10

**Authors:** Wm. W. Morgan


### SOLDERING MADE EASY.

THE usual method of soldering artificial dentures, as every practitioner well knows, is attended with no little labor and inconvenience. In view of the imperative call for some better means, and in the hope of overcoming, or at least mitigating these difficulties, I was led to contrive, that which I shall here designate, a DENTAL HAND FURNACE.

Notwithstanding I constructed and used this furnace nearly five years ago in the city of Philadelphia, it appears to be very little if *at all* known to the profession. And as I am ever willing to contribute my *mite* to promote the useful, I take this opportunity of assuring my professional friends, that it wholly supplies the *desideratum.*

MATERIAL, FORM AND DIMENSIONS.—The body of this furnace is made of Russia iron, No. 26. In form it is conical—its perpendicular depth is five inches—its top or greater diameter, five and a half inches—its base four inches, with an *unperforated bottom.* The top is finished by a No. 10 wire passing around it. The body for two inches above the bottom, is closely perforated with holes $\frac{1}{8}$ inch in diameter; close to the top of these holes, there is a sunken bead, or O. G. swage, which supports the grate. This grate should be made of No. 18 or 20 iron, also well perforated with holes $\frac{1}{4}$ inch in diameter. Over the groove or seam, and at the *bottom* of the furnace there is riveted a small iron shank, such as may be seen attached to the ordinary coal shovel found in every house. This shank lo ks downwards and outwards at an angle of 45 degrees, upon which is fitted a nicely turned wooden handle.

The space between the bottom of the furnace and the grate, serves a double purpose, first affording a good draft causing the fire to burn briskly when once under way, and secondly, not permitting the handle at any time to become uncomfortably warm.

Directions for use.—Fill the furnace with charcoal, and kindle; or with live wood coals if you have them. Your *teeth* having been previously put up in sand and plaster, you now place them on the burning coals, piling fresh coal all around the outside of the teeth. Do not use the *bellows* at first, as this would fracture the teeth, but after they have been on a few minutes, you may do so not only with impunity, but with advantage.

The plate by this means will be *gradually* heated up, the opposite of which is doubtless one great cause of the *plate springing.* As soon as the plate reaches a dull red heat, grasp the handle, which will allow you to wield the whole mass with great ease, carry it to your spirit-lamp, and with a mouth blow-pipe such as I have described in a previous number of the *Dental Register*, direct a pointed flame, and you will be surprised at the facility and rapidity with which this contrivance enables you to solder. I have frequently soldered by its aid a full arch in from two to three minutes, and under very favorable circumstances in a *single minute.* When the soldering is completed, lift off the teeth, empty the furnace, take out the grate, and place the teeth on it. Then turn the furnace upside down over them. This allows them to cool very gradually and uniformly, which will effectually prevent them from cracking, as well as afford another means of preventing the springing of the plate.

Sept. 5th, 1859. Wm. W. MORGAN.

## Figures and Tables

**Figure f1:**